# 134 Early Split-Thickness Autografting over a Collagen-Elastin Dermal Matrix Aids in Vascularization and Decreased Contraction

**DOI:** 10.1093/jbcr/irae036.133

**Published:** 2024-04-17

**Authors:** Heather M Powell, Britani Blackstone, Susannah Swanson, Naomi Mukka, Autumn Campbell, Zachary Everett, John Kevin Bailey

**Affiliations:** The Ohio State University, Columbus, Ohio; Wake Forest University, Winston Salem, NC; The Ohio State University, Columbus, Ohio; Wake Forest University, Winston Salem, NC; The Ohio State University, Columbus, Ohio; Wake Forest University, Winston Salem, NC; The Ohio State University, Columbus, Ohio; Wake Forest University, Winston Salem, NC; The Ohio State University, Columbus, Ohio; Wake Forest University, Winston Salem, NC; The Ohio State University, Columbus, Ohio; Wake Forest University, Winston Salem, NC; The Ohio State University, Columbus, Ohio; Wake Forest University, Winston Salem, NC

## Abstract

**Introduction:**

The dermis plays an important role in skin biomechanics and epidermal homeostasis. Following deep cutaneous injuries, restoration of the dermis is key for optimal functional and aesthetic outcomes. To regenerate a healthy, vascularized dermis, several dermal substitutes have been designed. The timing of split-thickness skin graft (STSG) application over these dermal substitutes is critical to success yet has not been studied systematically for some matrices.

**Methods:**

To understand the impact of STSG application timing, a collagen-elastin dermal matrix (CEDM) was applied to 2 x 1 in full-thickness acute surgical wounds created on the dorsum of red Duroc pigs following an IACUC approved protocol (10 sites per pig, 5 pigs). CEDMs were applied to the sites after which sites were grafted with meshed (1:1.5) and expanded STSG immediately or 4, 8 or 14 days following CEDM application. Sites without a CEDM beneath the STSG served as controls. Non-invasive measurements of tissue perfusion and contraction were collected at each site along with normal pig skin (NPS) at all timepoints with transepidermal water loss, pigmentation, erythema, contraction, and biomechanical properties quantified following grafting. Values were normalized to NPS for each pig. Biopsies (4mm) were collected and stained/immunostained to examine inflammation, blood vessel formation and dermal remodeling. All quantitative data were analyzed using a One-Way ANOVA with a Tukey posthoc test.

**Results:**

Engraftment rates were high when CEDM were grafted on D8 or earlier (> 97% engraftment); however, engraftment was more variable when CEDM were grafted on D14. There was no delay in re-establishment of epidermal barrier function as a result of the presence of the CEDM or as a function of application time. Four days post CEDM application, all groups were ~4-fold more perfused than normal pig skin. Following grafting, perfusion reduced to 2-fold NPS within one week of grafting. Perfusion within the CEDM remained high (4.5-5.2-fold NPS) until STSG application with the D14 group remaining at elevated perfusion levels through 6 weeks post-injury. Graft area was inversely related to time of grafting with CEDM sites grafted on D0 105.2% original area whereas sites grafted at day 14 post injury 70.5% original area (control sites 107.4% original area at week 12).

**Conclusions:**

In a porcine model, earlier application ( < 8 days) of STSGs over CEDMs was related to greater levels of engraftment and reduced levels of scar contraction. The CEDM became rapidly vascularized which may aid in the ability to be grafted immediately or soon after application.

**Applicability of Research to Practice:**

Data suggest immediate and early application of STSG over CEDMs does not result in graft loss or delayed re-epithelialization and may lead to improved outcomes.

